# Supplementary material for: An Objective Structured Clinical Exam on Breaking Bad News for Clerkship Students: In-Person Versus Remote Standardized Patient Approach
Source: MedEdPORTAL. 2023 Jul 21;19:11323. doi: 10.15766/mep_2374-8265.11323 (PMC10359437; doi:10.15766/mep_2374-8265.11323)
Supplement: Supplementary file 1 — SP Case.docxPatient Note.pdfPost-Follow-up Exercise.pdfPost-Follow-up Exercise Answer Key.docxSP Training Guide.pdfDoor Note (First Encounter).pdfDoor Note (Second Encounter).pdfSPIKES Protocol Checklist.pdfHistory Checklist.pdfFive-Question Survey.pdfOSCE Instructions.pdf [file mep_2374-8265.11323-s001.zip › G. Door Note (Second Encounter).pdf]

3. Door Note (Second Encounter)

MCO - AY2021 - Remote Clinical Curriculum - OBGYN - Nicky Granger - Pelvic Cramping (Blank Checklist)

Case Scenario

Clinical Setting:Emergency Room

Patient Information:
Name: Nicky Granger Age: 32 CC: Pelvic Pain and Bleeding
Vital signs: BP=95/68 RR=12 P= 98
Student Instructions:

You are rotating in the ER and have been asked to evaluate this patient via telemed portal.

You will haveten minutes to complete the following:

1. Based on the previous encounter and data provided formulate a diagnosis and management plan.
2. Deliver your diagnosis to the patient.
3. Provide counseling and reassurance to the patient.
4. Detail a management plan and next steps to patient.

Patient Exam ResultsPelvic exam – cervical os **open**, uterus anteverted, approximately 6cms, small amount of blood in vaginal vault.

Transvaginal sonogram – no gestational sac or embryo, normal fallopian tubes and ovariesbilaterally, endometrial lining 25mm – likely c/w retained embryonic tissue.

Blood Tests - CBC – hematocrit 35, white cell count 5, platelets 200, Beta HCG – 2200, type and screen is B negative, progesterone <5, thyroid stimulating hormone 2.4

No physical exam is necessary for this encounter.
