# Supplementary material for: An Objective Structured Clinical Exam on Breaking Bad News for Clerkship Students: In-Person Versus Remote Standardized Patient Approach
Source: MedEdPORTAL. 2023 Jul 21;19:11323. doi: 10.15766/mep_2374-8265.11323 (PMC10359437; doi:10.15766/mep_2374-8265.11323)
Supplement: Supplementary file 1 — SP Case.docxPatient Note.pdfPost-Follow-up Exercise.pdfPost-Follow-up Exercise Answer Key.docxSP Training Guide.pdfDoor Note (First Encounter).pdfDoor Note (Second Encounter).pdfSPIKES Protocol Checklist.pdfHistory Checklist.pdfFive-Question Survey.pdfOSCE Instructions.pdf [file mep_2374-8265.11323-s001.zip › H. SPIKES Protocol Checklist.pdf]

SPIKES Protocol Checklist

SPIKES: Breaking Bad News Protocol

(Modified SPIKES Protocol, Baile, W. et al. The Oncologist, 2000; 5:302-311)
The student did the following:

S - Setting up the Interview

1 Sat down.

- ◯ Yes
- ◯ No

2 Showed "listening" body language (eye contact, leaned forward, eye level).

- ◯ Yes
- ◯ No

P - Assessing Patient Perception

3 Asked me what I knew or had been told about the exam results.

- ◯ Yes
- ◯ No

I - Invitation

4 Asked me if I was ready to talk about the exam results/why I was in the office.

- ◯ Yes
- ◯ No

K - Giving Knowledge and Info to the Patient

5 Used language that I could understand (i.e. avoided technical terms).

- ◯ Yes
- ◯ No

6 Periodically assessed my understanding of what I was being told (asked me if I understood, asked me to repeat statements).

- ◯ Yes
- ◯ No

7 Corrected misunderstandings/misinformation.

- ◯ Yes
- ◯ No

8 Admitted to lack of knowledge when not equipped to answer question/did not have answer (e.g. was not able to state likelihood of future miscarriages).

- ◯ Yes
- ◯ No

E - Addressing Patient's Emotions with Empathic Responses

9 Gave me the opportunity to express my emotions.

- ◯ Yes
- ◯ No

10 Responded to me with empathy - eye contact, touched my arm, responses such as "I wish the news were better", or "I can understand that you would be upset."

- ◯ Yes
- ◯ No

S - Summary and Strategy

11 Discussed a management plan (e.g. I would need to have a D and C.)

- ◯ Yes
- ◯ No

SPIKES Protocol Checklist

12 **Asked me if I had any questions.**

- ◯ Yes
- ◯ No
