# Supplementary material for: An Objective Structured Clinical Exam on Breaking Bad News for Clerkship Students: In-Person Versus Remote Standardized Patient Approach
Source: MedEdPORTAL. 2023 Jul 21;19:11323. doi: 10.15766/mep_2374-8265.11323 (PMC10359437; doi:10.15766/mep_2374-8265.11323)
Supplement: Supplementary file 1 — SP Case.docxPatient Note.pdfPost-Follow-up Exercise.pdfPost-Follow-up Exercise Answer Key.docxSP Training Guide.pdfDoor Note (First Encounter).pdfDoor Note (Second Encounter).pdfSPIKES Protocol Checklist.pdfHistory Checklist.pdfFive-Question Survey.pdfOSCE Instructions.pdf [file mep_2374-8265.11323-s001.zip › I. History Checklist.pdf]

History Checklist

MCO - Class 2024 Clinical Curriculum - AY2022 - OBGYN - Nicky Granger - Pelvic Cramping (Blank Checklist)

History Checklist

The student asked me questions about the following:

1 Location of pain.

- ◯ Yes
- ◯ No

2 Quality of pain/Severity of pain.

- ◯ Yes
- ◯ No

3 Severity of pelvic bleeding?(e.g. how man pads have I bled through/ frequency of changing pads)

- ◯ Yes
- ◯ No

4 About any past medical problems, and for each diagnosis:

- ◯ Yes
- ◯ No

5 About past hospitalizations

- ◯ Yes
- ◯ No

6 About any past surgery, and why I had the surgery:

- ◯ Yes
- ◯ No

7 Do I take any medications, and if so the dose and frequency of each medication

- ◯ Yes
- ◯ No

8 Do I have any allergies

- ◯ Yes
- ◯ No

9 What was the specific allergic reaction

- ◯ Yes
- ◯ No

10 Do I take any supplements and/or use any form of complementary medicine

- ◯ Yes
- ◯ No

11 Am I currently sexually active

- ◯ Yes
- ◯ No

12 Are my partners male, female, or both

- ◯ Yes
- ◯ No

13 Do I have vaginal sex, oral sex, and/or anal sex

- ◯ Yes
- ◯ No

14 Do I/does my partner use any form of contraception

- ◯ Yes
- ◯ No

15 Have I ever had a sexually transmitted disease, pelvic inflammatory disease or ovarian cysts.

- ◯ Yes
- ◯ No

16 Any history of fibroids, endometriosis or any abnomal pap smears.

- ◯ Yes
- ◯ No

17 Are my periods regular and how long do they last

- ◯ Yes
- ◯ No

18 When was my last menstrual period

- ◯ Yes
- ◯ No

19 Have I ever been pregnant, and if I have been pregnant: number of deliveries, about any complications related to pregnancy

- ◯ Yes
- ◯ No

20 If I have ever had a miscarriage and if so did it require a dilation and curettage procedure

- ◯ Yes
- ◯ No

21 Timing of first miscarriage (How many weeks were you pregnant before miscarrying?)

- ◯ Yes
- ◯ No

22 Any history of hypothyroidism and it's treatment.

- ◯ Yes
- ◯ No

23 Any history of anxiety and it's treatment.

- ◯ Yes
- ◯ No

24 Do my parents, siblings or children have any medical problems

- ◯ Yes
- ◯ No

H. The Review of Systems (ROS) (Must ask at least 2)

25 General/systemic - Fever, chills, night sweats, any changes in weight, any changes in appetite, fatigue

- ◯ Yes
- ◯ No
