# Supplementary material for: An Objective Structured Clinical Exam on Breaking Bad News for Clerkship Students: In-Person Versus Remote Standardized Patient Approach
Source: MedEdPORTAL. 2023 Jul 21;19:11323. doi: 10.15766/mep_2374-8265.11323 (PMC10359437; doi:10.15766/mep_2374-8265.11323)
Supplement: Supplementary file 1 — SP Case.docxPatient Note.pdfPost-Follow-up Exercise.pdfPost-Follow-up Exercise Answer Key.docxSP Training Guide.pdfDoor Note (First Encounter).pdfDoor Note (Second Encounter).pdfSPIKES Protocol Checklist.pdfHistory Checklist.pdfFive-Question Survey.pdfOSCE Instructions.pdf [file mep_2374-8265.11323-s001.zip › J. Five-Question Survey.pdf]

Five Question Survey

Please respond:

1 The learning objectives for the session were clear.

- ◯ Strongly Agree
- ◯ Agree
- ◯ Neutral
- ◯ Disagree
- ◯ Strongly Disagree

2 The amount of preparation material was just about right.

- ◯ Strongly Agree
- ◯ Agree
- ◯ Neutral
- ◯ Disagree
- ◯ Strongly Disagree

3 The instructor summarized the most important points.

- ◯ Strongly Agree
- ◯ Agree
- ◯ Neutral
- ◯ Disagree
- ◯ Strongly Disagree

4 I learned something that will be useful in caring for gynecology patients.

- ◯ Strongly Agree
- ◯ Agree
- ◯ Neutral
- ◯ Disagree
- ◯ Strongly Disagree

5 This was a worthwhile educational experience for me.

- ◯ Strongly Agree
- ◯ Agree
- ◯ Neutral
- ◯ Disagree
- ◯ Strongly Disagree

6 Comments:
