# Supplementary material for: An Objective Structured Clinical Exam on Breaking Bad News for Clerkship Students: In-Person Versus Remote Standardized Patient Approach
Source: MedEdPORTAL. 2023 Jul 21;19:11323. doi: 10.15766/mep_2374-8265.11323 (PMC10359437; doi:10.15766/mep_2374-8265.11323)
Supplement: Supplementary file 1 — SP Case.docxPatient Note.pdfPost-Follow-up Exercise.pdfPost-Follow-up Exercise Answer Key.docxSP Training Guide.pdfDoor Note (First Encounter).pdfDoor Note (Second Encounter).pdfSPIKES Protocol Checklist.pdfHistory Checklist.pdfFive-Question Survey.pdfOSCE Instructions.pdf [file mep_2374-8265.11323-s001.zip › K. OSCE Instructions.pdf]

OSCE Instructions:

Introduction to OSCE

1. A brief overview with OSCE evaluator in the main room **(5 minutes)**
2. Assumption: Clinical setting is a remote encounter with the patient in the ER

Remote encounter via Zoom

1. Encounter in the breakout room: Obtain a history, mention system exams you would want to perform and specifically describe steps of a pelvic exam to your standardized patient (SP) **(15 minutes)**
2. Results in the main room: Receive data on pelvic exam findings, sonogram and blood test values **(5 minutes)**
3. Encounter in the breakout room: Deliver bad news to the SP **(10 minutes)**
4. After 10 minutes has passed, SP's camera turns off, stay in the breakout room for SP feedback **(5 minutes)**
5. Back to main room for debriefing with OSCE evaluator **(10 minutes)**
6. Complete **patient note** and **post-follow up quiz** and email to clerkship coordinator at end of the OSCE
