# Supplementary material for: An Objective Structured Clinical Exam on Breaking Bad News for Clerkship Students: In-Person Versus Remote Standardized Patient Approach
Source: MedEdPORTAL. 2023 Jul 21;19:11323. doi: 10.15766/mep_2374-8265.11323 (PMC10359437; doi:10.15766/mep_2374-8265.11323)
Supplement: Supplementary file 1 — SP Case.docxPatient Note.pdfPost-Follow-up Exercise.pdfPost-Follow-up Exercise Answer Key.docxSP Training Guide.pdfDoor Note (First Encounter).pdfDoor Note (Second Encounter).pdfSPIKES Protocol Checklist.pdfHistory Checklist.pdfFive-Question Survey.pdfOSCE Instructions.pdf [file mep_2374-8265.11323-s001.zip › A. SP Case.docx]

Appendix A: *MedEdPORTAL* Standardized Patient Case Development Tool

Date: July 15, 2022

Primary Case Author: Lona Prasad MD

Secondary Case Author: Steven Hockstein MD

Standardized Patient Educator: Kevaughn Harvey

Name of Case: Breaking Bad News in a Virtual Setting: A Standardized Patient Miscarriage Telemedicine Objective Structured Clinical Exam

Name of Educational and/or Assessment Activity: Telemedicine miscarriage OSCE session

Patient Name: Nicky Granger

Chief Complaint: Heavy vaginal bleeding and positive urine pregnancy test

Most Likely Diagnosis and Differential With Rationale From History and/or Physical Exam: Early pregnancy loss – Incomplete miscarriage. Differentials (from History): Ectopic pregnancy, Inevitable miscarriage, Complete miscarriage, Threatened miscarriage

Challenge Question: Taken from the Patient Note; List up to 3 diagnoses that might explain the patient’s complaint(s), from most likely to least likely. Then enter the positive or negative findings from the history and the physical examination (if present) that support each diagnosis. Lastly, list initial diagnostic studies you would order for each listed diagnosis.

Domains: Check all that apply

X Professionalism

X Communication and Interpersonal Skills

X Medical History

- Physical Exam

X Shared Decision-Making

X Patient Education

X Clinical Reasoning

X Documentation

Handoff

X Presentation

X Other: Breaking Bad News

Type and Level of Learner: Medical Student – Second and third year medical students

Case Objectives: Please list specific objectives for each of the domains you have checked above:

1. Demonstrate professionalism in the domains of respect and compassion when breaking bad news

2. Interview, clinically assess and provide management options to a standardized patient having a miscarriage

3. Incorporate the SPIKES protocol while communicating with the patient

4. Completion of the Patient Note and Post Follow Up Exercise

| SETTING: outpatient, in patient, ED, home, nursing home, rehab, group, etc. | Patient in the ED having the telehealth visit with the student located remotely |
| --- | --- |
| PATIENT PROFILE: Information about the “patient” that helps select an SP and helps the learner get an understanding of them as a person. SP will know more information about the patient than learner will ever ask but allows SP to portray a fully developed patient personality. If none of the items below are particulars for the case, please write “all may be used.” | |
| Age range | 32 years old |
| Religious/spiritual background | all may be used |
| Sex (e.g., male, female, intersex, transwoman, transman) | female |
| Sexual orientation (e.g., heterosexual, lesbian, gay, bisexual, pansexual, queer, asexual) | heterosexual |
| Gender expression (e.g., man, woman, genderqueer) | woman |
| Race and ethnicity | all may be used |
| Physical description (e.g., BMI, height range) | all may be used |
| Physical limitations | none |
| Patient appearance (e.g., disheveled, hospital gown, business casual, casual) | hospital gown for the in-person OSCE, street clothes for the remote OSCE |
| Moulage + location (e.g., none, bruises, scars, body piercing, tattoos) | all may be used |
| Affect (e.g., pleasant, cooperative) | pleasant, cooperative, mildly upset and anxious |
| Family group (e.g., who is family, who they live with) | lives with husband |
| Education | teacher education degree |
| Level of health literacy | all may be used |
| Employment, if any - present and past, noting any current stresses | elementary school science teacher |
| Home/homeless - type of dwelling, number of stories, owned or rented | home – apartment in the upper east side of New York |
| Financial situation - any current stresses | no financial stresses – husband is a business consultant |
| Insurance status (e.g., un/under/insured, public/private, HMO/PPO) | private |
| Habits (i.e., diet, exercise, caffeine, smoking, alcohol, drugs) | eating take out foods, working out less than usual, non-smoker, has 7 alcoholic drinks per week, never used recreational drugs |
| Activities (i.e., hobbies, sports, clubs, friends) | all may be used; however less involved in hobbies and interests as patient is often tired from the early and long days at school |
| Typical day - what is the usual daily routine | goes to work daily at the elementary school as a science teacher during the weekdays |

| CASE INFORMATION | |
| --- | --- |
| Chief Concern: What the patient will say when greeted by the student. The patient’s primary reason for seeking medical care often stated in their own words. | I’m having really bad pelvic pain and I can’t stop bleeding. |
| Additional Concerns: Other, if any, concerns the patient has today (i.e., symptoms, requests, expectations, etc.) that will become part of set agenda. | Had a positive home pregnancy test and experienced similar symptoms of bleeding after her previous miscarriage. Afraid she is having a miscarriage again. |
| THE PATIENT’S STORY: The SP will be asked to tell their symptom story and the personal and emotion impact for each of their concerns. You will want to write this in the patient’s voice. The symptom story should be able to answer this question: “Tell me more about [chief concern/additional concern], starting at the beginning and bringing me up to now.”  The personal context should be able to answer questions concerning the broader personal/psychosocial context of symptoms, especially the patient’s beliefs/attributions.  The emotional context should be able to ask how are you doing with this, how does this make you feel, how has this affected you emotionally? IMPACT: How has this affected your life? How has this been for your family? | Since my period was not on time, I decided to take a home pregnancy test which was positive. A few hours later I started having painful pelvic cramps and heavy bleeding with clots. I called my gynecologist’s office and spoke with a nurse who advised me to come to the ER. I’ve been bleeding now for the past 4 hours. I’ve soaked through 4-5 maxi pads and my clothes. The pain has gotten worse, like an awful period, especially when I’m passing clots. The clots are the size of ping pong balls and are darkish red. This is really stressful. I’m worried I’m having another miscarriage. I’ve been so busy and tired lately and barely see my husband who is traveling a lot for work. We have been wanting to have a baby for awhile now. I do feel pressure from my husband’s siblings who all have children. All of this is making me overwhelmed (SP is visibly upset, crying, at times has shallow breathing). |
| HISTORY OF PRESENT ILLNESS: Although some of the HPI will be given in the patient’s symptom story, the learners will expand the story during the direct question section. Below, describe the detailed history, usually about the chief concern, which the student must develop in order to make a useful assessment of the problem: | |
| Onset (when; gradual or sudden) | Bleeding and pelvic pain started slowly and gradually became stronger. |
| Setting (what was going on or where was patient when symptoms first noticed?) | Patient was at home when symptoms started. |
| Duration (how long) | Was experiencing symptoms at home for 4 hours. |
| Time relationships (frequency, constant or intermittent) | Once symptoms started, stayed constant, with intermittent bursts of worsening cramping pelvic pain and passage of large blood clots. |
| Location | Pelvic |
| Radiation | Into the vagina and lower back |
| Quality | Cramping, throbbing |
| Amount | Pain is on the scale of 6 or 7 and spikes to 8 or 9 when passing a clot. |
| Aggravated by what | Not applicable |
| Relieved by what | Nothing |
| Associated with what | Worsening pain associated with passage of large clots |
| Attitude (what does the patient think is the problem, and how do they feel about it) | Patient thinks she is having a miscarriage. She feels anxious, upset and sad. |
| Overall course | Symptoms are worsening. Patient denies lightheadedness or heart palpitations. |
| REVIEW OF SYSTEMS: Significant positives and negatives | |
| Constitutional - negative | Genito-urinary - negative |
| HEENT – negative | Musculoskeletal - negative |
| Cardiovascular – negative | Skin/breast - negative |
| Respiratory - negative | Neurological - negative |
| Gastroenterology - **constipation** | Psychiatric - **anxiety** |
| Past medical history |  |
| Medication allergies (name and reaction) | None |
| Environmental allergies (name and reaction) | None |
| Illnesses | Hypothyroidism, anxiety |
| Vaccinations | Up to date with routine vaccinations |
| Surgeries | Hysteroscopic resection of submucosal fibroid, dilation and curettage for miscarriage management |
| Accidents/injuries/trauma | None |
| Hospitalization | None |
|  | |
| Inclusive sexual and reproductive history | |
| Sexual practices  Sexual partners  Protection: Use of safer sex practices  Use of birth control if appropriate  Risk of intimate partner violence | Monogamous with husband, penetrative sexual intercourse  Three lifetime sexual partner including spouse. Had one female partner, however, identifies as heterosexual. No use of contraception as been trying to conceive. No risk of intimate partner violence. |
| OB/GYN history | Age of onset of menses: 12  Age of menopause: not applicable  Number of pregnancies: 2  Number of live births: none  Number of miscarriages: 1 (not including the present case)  Number of abortions: 0 |
| Medications | Prescription/dose/reason: synthroid/50mcgs/hypothyroidism  Over the counter/dose/reason: none  Herbs/supplements/dose/reason: none  Other: none |
| Immunizations | X Tetanus  X Flu  X Hepatitis  X Pneumovax  X HPV  X COVID |
| Tobacco products:   - Cigarettes - Cigar - Pipe - Chew - E-cigarettes | X Never   - Past - year started/year quit - Current   - Quantity   - # of years |
| Alcohol   - Beer   X Wine   - Liquor - Other | - Never - Past - year started/year quit - Current   - Quantity   - # of years |
| Drugs   - Weed - Cocaine - Heroin - Meth - IV - Inhalants - Other | X Never   - Past - year started/year quit - Current   - Quantity   - # of years |
| Diet (describe) | Take out foods – Chinese, Indian, Japanese, Italian |
| Exercise (describe) | Less than usual; takes short evening walks if isn’t tired |
| List any other important social history or information important to this case | Diagnosed with anxiety, saw a psychiatrist who recommended anxiolytics. Patient declined as she was trying to conceive and didn’t feel comfortable. Also recommended yoga and meditation, however, patient was not interested in pursuing this. |
| Family history |  |
| Mother, father, siblings, grandparents, and other significant findings | Mother diagnosed with breast cancer at age 62; in remission. Father has hypertension which is well controlled. Sister has endometriosis and had difficulty conceiving. |
|  |  |
| Physical Exam - List exam maneuvers expected for this case and any abnormal findings that SP will simulate. (tenderness, hyper-hypo reflex, rebound, weakness, etc.)  In-person OSCE:  Expected to examine neck for thyroid gland, respiratory and cardiac systems which are all normal.  Abdomen – diffuse pain in the lower pelvic area.  In-person and telemedicine OSCE:  Medical student given a cue by the patient to describe the steps of a pelvic examination. | |
| PHYSICAL EXAM FINDINGS |  |
| 1. Written in layperson’s terms | Painful abdomen |
| 1. General appearance - affect, appearance, position of patient at opening (i.e., sitting, lying down, holding abdomen, etc.) | Visibly upset, crying, at times has shallow breathing. Sitting up and holding abdomen. |
| 1. Vital signs | BP=95/68, RR=12, P=98 |
| 1. Specific findings and affect | Tenderness in the lower abdomen and pelvis. Student is provided with pelvic exam findings which includes an open cervical os and small amount of blood in the vaginal vault. |
| 1. Response to certain physical movements | Uncomfortable upon abdominal palpation |
|  |  |
| DIAGNOSIS AND DIFFERENTIAL |  |
| Diagnosis with support from positive and negative history and PE findings | Incomplete miscarriage presented with cramping pelvic pain, vaginal bleeding with clots and positive urinary pregnancy test. Exam consistent with blood in the vaginal vault and open cervical os. |
| Differential with support from positive and negative history and PE findings | Ectopic pregnancy, inevitable, complete and threatened miscarriages can present with cramping pelvic pain, vaginal bleeding and a positive urinary pregnancy test. However with ectopic pregnancy, complete and threatened miscarriages on pelvic examination there may be blood in the vaginal vault, however, the cervical os is closed. The cervical os is open in an inevitable miscarriage, however, unlike an incomplete miscarriage, all conception products are seen in the uterus on sonogram. |
|  |  |
| MANAGEMENT OR DIAGNOSTIC PLAN | Patient given option for observation, medical management with vaginal cytotec or surgery with dilation and curettage. |
|  |  |
| PROFESSIONALISM ISSUES OR CHALLENGES | Navigating the zoom set up alternating between the main and breakout rooms can be confusing occasionally for some of the students. |
