# Supplementary material for: An Objective Structured Clinical Exam on Breaking Bad News for Clerkship Students: In-Person Versus Remote Standardized Patient Approach
Source: MedEdPORTAL. 2023 Jul 21;19:11323. doi: 10.15766/mep_2374-8265.11323 (PMC10359437; doi:10.15766/mep_2374-8265.11323)
Supplement: Supplementary file 1 — SP Case.docxPatient Note.pdfPost-Follow-up Exercise.pdfPost-Follow-up Exercise Answer Key.docxSP Training Guide.pdfDoor Note (First Encounter).pdfDoor Note (Second Encounter).pdfSPIKES Protocol Checklist.pdfHistory Checklist.pdfFive-Question Survey.pdfOSCE Instructions.pdf [file mep_2374-8265.11323-s001.zip › B. Patient Note.pdf]

Patient Note

PATIENT NOTE

1 HISTORY: Describe the history you just obtained from this patient. Include only information (pertinent positives and negatives) relevant to this patient's problem(s).

2 PHYSICAL EXAMINATION: Describe any positive and negative findings relevant to this patient's problem(s). Be careful to include ONLY those parts of examination you performed in THIS encounter.

Data Interpretation: Based on what you have learned from the history and physical examination, list up to 3 diagnoses that might explain this patient's complaint(s). List your diagnoses from most likely to least likely. For some cases, fewer than 3 diagnoses will be appropriate. Then, enter the positive or negative findings from the history and the physical examination (if present) that support each diagnosis. Lastly, list initial diagnostic studies (if any) you would order for each listed diagnosis (e.g. restricted physical exam maneuvers, laboratory tests, imaging, ECG, etc.).

3 Diagnosis #1: List diagnosis as well as positive or negative findings from the history and the physical examination that support your diagnosis.

4 Diagnosis #2: List diagnosis as well as positive or negative findings from the history and the physical examination that support your diagnosis.

Patient Note

5 **Diagnosis #3: List diagnosis as well as positive or negative findings from the history and the physical examination that support your diagnosis.**

6 **Diagnostic Studies: List initial diaganostic studies (if any) you would order for each listed diagnosis.**
