# Supplementary material for: An Objective Structured Clinical Exam on Breaking Bad News for Clerkship Students: In-Person Versus Remote Standardized Patient Approach
Source: MedEdPORTAL. 2023 Jul 21;19:11323. doi: 10.15766/mep_2374-8265.11323 (PMC10359437; doi:10.15766/mep_2374-8265.11323)
Supplement: Supplementary file 1 — SP Case.docxPatient Note.pdfPost-Follow-up Exercise.pdfPost-Follow-up Exercise Answer Key.docxSP Training Guide.pdfDoor Note (First Encounter).pdfDoor Note (Second Encounter).pdfSPIKES Protocol Checklist.pdfHistory Checklist.pdfFive-Question Survey.pdfOSCE Instructions.pdf [file mep_2374-8265.11323-s001.zip › E. SP Training Guide.pdf]

SP TRAINING GUIDE:
Nicky Granger

Students: Third year medical students

Student Learning Objectives for Case:

1. Students will be able to conduct a focused history and describe physical examination steps in a manner comfortable to the patient.
2. Students will be able to generate a differential diagnosis list for a chief complaint of pelvic cramps and bleeding.
3. Students will be able to communicate clearly an initial diagnosis and management plan to the patient.
4. Student will be able to deliver bad news with empathy and provide reassurance to a patient as part of their training.

Student skills standardized patient will be observing/assessing (outlined in detail on checklists):

1. Breaking bad news and communication skills (using SPIKEs protocol)
2. History taking (using History checklist)

Wardrobe: You will be in street clothes

Makeup: Little to no makeup

Demeanor/Appearance: You are in pain from the pelvic cramps and very anxious and worried that you may be having a miscarriage. You are visibly upset, sometimes crying, and at times you have shallow breathing. Despite this you are cooperative and are willing to answer the student doctor's questions if it'll help figure out what's happening to you.

General Guidelines:

1. There is some medical information in the **History of Chief Complaint section** that you will provide only if you are specifically asked by the student. This information appears under the heading **"If the student specifically asks."**

2. You should only provide other medical information like your past medical problems and habits such as smoking or drinking **if you are specifically asked**.

3. If the student asks you about a symptom or medical problem that has not been included in this guide, please respond that you **don't have the symptom and/or you don't have that medical problem.**

Opening Statement for Standardized Patient: "I'm having really bad pelvic pain and I can't stop bleeding."

Response to initial open-ended question/information that the SP will volunteer:

HPI:
Since your period was not on time you decided to take a home pregnancy test and discovered that you were pregnant with a positive UPT. A few hours later you started experiencing painful pelvic cramps and heavy bleeding with clots. You called your doctor's office and spoke with a nurse who advised you to come in to be seen by a physician.

You've been experiencing heavy bleeding with large clots for the past four hours. The bleeding is soaking through maxi pads. Along with this you've had cramping pelvic pain. You are still currently bleeding and in pain. The pain is constant but not overpowering but it does spike intermittently when you are passing a blood clot.

Past ObGyn history:
- **Ob Hx:** Two years ago first trimester miscarriage; bled heavily, required dilation and curettage surgery. Your first miscarriage was characterized by pelvic pain/heavy bleeding. At the time, your doctor said that your cervix was open and that you needed surgery to stop the bleeding and remove the remainder of the fetus. Your current symptoms are very reminiscent of your first miscarriage and you're worried that it's happening all over again. **(Please wait for student to ask follow-up questions before giving details of miscarriage)**

Gyn Hx: Menses – normal, bleeds q28 days, 4-5 days. Sexually active with spouse, not using contraception as you are trying to conceive. LMP: 6 weeks ago
You have a history of fibroids **(student should ask what kind of fibroids and if and how treated)**

No history of STD/PID/ovarian cysts/endometriosis/abnormal pap smear. Last pap smear was one year ago.

If the student specifically asks items:
- You've had no associated lightheadedness or heart palpitations.

- Pain is on the scale of 6 or 7 at baseline and spikes higher 8 or 9 when passing a clot. If they don't use the pain scale the cramping is "like a really bad period cramp.

- Clot size is about the size of a ping pong ball. Color is darkish red.

- You have no other discharge.

- You have bled through 4 to 5 pads already. Changing one out every hour. The bleeding was also soaking through your clothes.

- Your first miscarriage happened when you were eight weeks pregnant. You had similar symptoms and went to the ED.

- Fibroid Details: Submucosal fibroids (embedded in endometrial lining) Diagnosed four years ago after ultrasound. You saw your gynecologist due to abnormally heavy bleeding and painful periods. You also experienced bleeding between periods.
    - Treatment involved surgery to remove fibroid by “hysteroscopy” – minimally invasive surgery with a camera entering through the vagina.

Past medical history:
Hypothyroidism – Diagnosis by primary care doctor five years ago (Symptoms – routinely cold/sluggish/heavy periods

Past surgeries:
Minimally invasive fibroid surgery (See fibroid details)
“Scraping in OR after first pregnancy.”

Medications and supplements:
Synthroid for the hypothyroidism – 50 micrograms
Pre-natal vitamins

Allergies: None

Social history:
You are a 32-year old elementary school science teacher working at a private school on the UES. You live with your husband, a business consultant, in Yorkville. You’ve been married for five years. Your husband travels a lot for work and you are often tired from the early and long days at school so you are not as active and involved in your community/hobbies/interests as you’d like to be. You have some anxiety surrounding your marriage and having a baby. Your husband comes from a large family, the youngest of six. All his siblings have children. Your in-laws keep asking when you’re “going to add to the family.”

<u>Diet/Exercise:</u> You haven’t been working out as much and tend to order takeout most nights (you don’t want to go to the trouble of cooking just for yourself). You also have been working out less since you’ve started the school year. You would like to exercise more and eat healthier but “life gets in the way.” You feel that you’ve put on a few pounds recently but you’re not terrible concerned about it.

<u>Alcohol:</u> seven drinks/week - usually wine with dinner.

<u>Smoking:</u> None. No past smoking

<u>Drugs:</u> None. No past drug use.

Sexual History: See Gyn Hx above. Three lifetime sexual partners including your spouse. One female partner though you identify as "straight." Post-miscarriage you waited three months until you were cleared by your doctor to try to get pregnant again. You and your partner are actively trying to conceive but it's been challenging given your work schedules. It's a source of stress for you.

Family history:
Mother – 65 y/o. Diagnosed with breast cancer age 62. Caught early. She was treated and in remission.
Father – 66 y/o – Hypertension. Controlled as far as you know.
Sister – 35yo --Endometriosis. She has one child. You know it was tough for her to get pregnant. Beyond that you don't have a lot of details but you are aware that she has received treatment for it.

ROS:
Constitutional – negative
HEENT – negative
Cardiovascular – negative
Respiratory – negative
Gastroenterology – **Constipation –** *Bowel movements only 3x a week instead of everyday. Stools are dry and difficult to pass. Coffee and hot water help. Mostly controlled now that you're on Synthroid. Not a primary concern.*
Genito-urinary – negative
Musculoskeletal – negative
Skin/breast – negative
Neurological – negative
Psychiatric – **Anxiety –** *Primary Care Doctor recommended you see a Psychiatrist. You were reluctant at first but decided to give it a try. Diagnosed with anxiety two months ago by psychiatrist. You declined meds because you're hoping to get pregnant and didn't want to be on psychiatric medication at the same time. If asked, you can't remember what medication was suggested. The psychiatrist also recommended yoga/meditation but you were resistant because it sounded "**too new-agey**"*

<u>At this point the student should transition to the Physical Exam. Note that the student will not be conducting a systems exam or pelvic/genitourinary exam. Students should comment that they would do the following exams if in-person.</u>

<u>Physical Exam:</u>
General demeanor: **Visibly upset, crying, at times has shallow breathing**
Neck: would check your thyroid for enlargement by placing hands on the back of your neck.
Respiratory: would check lungs in the back/front with a stethoscope.
Cardiac: would listen to the heart with a stethoscope.
Abdomen: would press and listen to your abdomen with a stethoscope.

Student would then describe to the patient the steps of a pelvic exam to include:

Pelvic:
- Check external genitalia and urethra.
- Place speculum in vagina, look at vagina and cervix for signs of trauma and/or lesions.
- **Check if** the cervix open.
- **Check for** active bleeding or blood in the vagina
- Check if cervical is open or closed.
- Confirm if uterus mobile, examine for size, is it anteverted, retroverted or midaxial? Is there adnexal tenderness or masses bilaterally?

At this point you will thank the student for describing the exam and ask the student to give you some time alone. Student will then leave the breakout room to complete their patient note in the main room.

Based on the Hx and exam student should come up with the following diagnoses:

Differential Diagnoses: *Miscarriage – threatened, inevitable, incomplete, complete or ectopic pregnancy, cervical or uterine polyp, gestational trophoblastic disease (extra points).*
Tests to order: *transvaginal sonogram and blood tests – CBC, beta HCG, type and screen, progesterone (extra point), thyroid stimulating hormone (extra point).*

Then Student is given correct answers to fit this scenario:

Pelvic exam *– cervical os closed, uterus anteverted, approximately 6cms, small amount of blood in vaginal vault.*
Transvaginal sonogram *– no gestational sac or embryo, normal fallopian tubes and ovaries bilaterally, endometrial lining 25mm – likely c/w retained embryonic tissue.*
Blood Tests *- CBC – hematocrit 35, white cell count 5, platelets 200,*
beta HCG – 2200, type and screen is B negative, progesterone <5, thyroid stimulating hormone 2.4
(Student given a few minutes to formulate diagnosis and assessment and plan)

Encounter 2*:*
They will then have up to 10 minutes to return to room and give diagnosis/break bad news and provide assessment and plan to the patient:

Student prompted to return to room and give diagnosis/break bad news and provide assessment and plan to the patient:
You are 6 weeks pregnant, bleeding heavily and experiencing pelvic pain.
You have had an incomplete miscarriage. This is not a viable pregnancy, so you have passed most of the fetus however products of conception are still in the uterus.

Upon hearing the news, you are very upset upset/crying. And student is meant to provide support/reassurance.

And then discusses management options:
Medication oriented (cytotec) vs surgical (dilation and curettage) – Student should be able to describe both options and associated risks of each (i.e. failure with cytotec, need for d&c, risk for ashermans syndrome; with medical management could have heavy bleeding, anemia.

Once you're informed of your options let the student doctor know that you would like some time to decide on this and will call the physician after discussing with partner.

One week prior the students will be encouraged to read 4 specific articles in preparation for this OSCE.
