# Supplementary material for: An Objective Structured Clinical Exam on Breaking Bad News for Clerkship Students: In-Person Versus Remote Standardized Patient Approach
Source: MedEdPORTAL. 2023 Jul 21;19:11323. doi: 10.15766/mep_2374-8265.11323 (PMC10359437; doi:10.15766/mep_2374-8265.11323)
Supplement: Supplementary file 1 — SP Case.docxPatient Note.pdfPost-Follow-up Exercise.pdfPost-Follow-up Exercise Answer Key.docxSP Training Guide.pdfDoor Note (First Encounter).pdfDoor Note (Second Encounter).pdfSPIKES Protocol Checklist.pdfHistory Checklist.pdfFive-Question Survey.pdfOSCE Instructions.pdf [file mep_2374-8265.11323-s001.zip › F. Door Note (First Encounter).pdf]

1. Door Note (First Encounter)

MCO - AY2021 - Remote Clinical Curriculum - OBGYN - Nicky Granger - Pelvic Cramping (Blank Checklist)

Case Scenario

Clinical Setting: Emergency Room

Patient Information:
Name: Nicky Granger
Age: 32 CC: Pelvic Pain and Bleeding
Vital signs: BP=95/68 RR=12 P= 98
Student Instructions:

You are rotating in the ER and have been asked to evaluate this patient via telemed portal.

You will have 15 minutes to complete the following:

1. **Focused history**
2. **Focused PE**
3. **Description of pelvic exam steps to patient/faculty**
