# Supplementary material for: An Objective Structured Clinical Exam on Breaking Bad News for Clerkship Students: In-Person Versus Remote Standardized Patient Approach
Source: MedEdPORTAL. 2023 Jul 21;19:11323. doi: 10.15766/mep_2374-8265.11323 (PMC10359437; doi:10.15766/mep_2374-8265.11323)
Supplement: Supplementary file 1 — SP Case.docxPatient Note.pdfPost-Follow-up Exercise.pdfPost-Follow-up Exercise Answer Key.docxSP Training Guide.pdfDoor Note (First Encounter).pdfDoor Note (Second Encounter).pdfSPIKES Protocol Checklist.pdfHistory Checklist.pdfFive-Question Survey.pdfOSCE Instructions.pdf [file mep_2374-8265.11323-s001.zip › C. Post-Follow-up Exercise.pdf]

Student Post-Followup Exercise

STUDENT EXERCISE

Causes for miscarriage include:

1 Chromosonal abnormalities.

- ◯ True
- ◯ False

2 Young Age

- ◯ True
- ◯ False

3 Diabetes Mellitus

- ◯ True
- ◯ False

4 Fibroids

- ◯ True
- ◯ False

This patient can reduce the chances of miscarriage again by:

5 Reducing Alcohol intake

- ◯ True
- ◯ False

6 Starting Anxiolytics

- ◯ True
- ◯ False

7 Gaining Weight

- ◯ True
- ◯ False

8 Stress reduction

- ◯ True
- ◯ False

Choose the best Answer:

9 What type of thrombophilia is most likely to be associated with recurrent pregnancy loss?

- ◯ Factor V Leiden
- ◯ Protein C Deficiency
- ◯ Antiphospholipid Syndrome
- ◯ Protein S Deficiency

10 What is the definition of Recurrent Pregnancy Loss?

- ◯ Greater than 1 miscarriage
- ◯ 2 or more miscarriages
- ◯ Greater than 3 miscarriages
