# Supplementary material for: An Objective Structured Clinical Exam on Breaking Bad News for Clerkship Students: In-Person Versus Remote Standardized Patient Approach
Source: MedEdPORTAL. 2023 Jul 21;19:11323. doi: 10.15766/mep_2374-8265.11323 (PMC10359437; doi:10.15766/mep_2374-8265.11323)
Supplement: Supplementary file 1 — SP Case.docxPatient Note.pdfPost-Follow-up Exercise.pdfPost-Follow-up Exercise Answer Key.docxSP Training Guide.pdfDoor Note (First Encounter).pdfDoor Note (Second Encounter).pdfSPIKES Protocol Checklist.pdfHistory Checklist.pdfFive-Question Survey.pdfOSCE Instructions.pdf [file mep_2374-8265.11323-s001.zip › D. Post-Follow-up Exercise Answer Key.docx]

Student Post-Follow-up Exercise Answer Key

STUDENT EXERCISE

**Causes for miscarriage include:**

Chromosomal abnormalities – True

Young age – False

Diabetes Mellitus – True

Fibroids – True

**This patient can reduce the chances of miscarriage again by:**

Reducing Alcohol Intake – True

Starting Anxiolytics – False

Gaining Weight – False

Stress reduction – True

**Choose the best answer:**

What type of thrombophilia is most likely to be associated with recurrent pregnancy loss?

Antiphospholipid Syndrome

What is the definition of Recurrent Pregnancy Loss?

2 or more miscarriages
