# Supplementary material for: An Objective Structured Clinical Exam on Breaking Bad News for Clerkship Students: In-Person Versus Remote Standardized Patient Approach
Source: MedEdPORTAL. 2023 Jul 21;19:11323. doi: 10.15766/mep_2374-8265.11323 (PMC10359437; doi:10.15766/mep_2374-8265.11323)
Supplement: Supplementary file 1 — SP Case.docxPatient Note.pdfPost-Follow-up Exercise.pdfPost-Follow-up Exercise Answer Key.docxSP Training Guide.pdfDoor Note (First Encounter).pdfDoor Note (Second Encounter).pdfSPIKES Protocol Checklist.pdfHistory Checklist.pdfFive-Question Survey.pdfOSCE Instructions.pdf [file mep_2374-8265.11323-s001.zip › Educational Summary Report_11323.pdf]

MedEdPORTAL® The AAMC Journal of Teaching and Learning Resources

Original Publication Open Access

An Objective Structured Clinical Exam on Breaking Bad News for Clerkship Students: In-Person Versus Remote Standardized Patient Approach

Lona Prasad, MD*, Steven Hockstein, MD, Joseph E. Safdieh, MD, Kevaughn Harvey, MFA, Paul J. Christos, DrPH, Yoon Kang, MD

*Corresponding author: lop9006@med.cornell.edu

Abstract

Introduction: Telemedicine training for medical students is critical as that modality becomes integral to patient care. This formative standardized patient (SP) objective structured clinical exam (OSCE) lets students discuss miscarriage diagnosis and treatment virtually. **Methods:** The SP OSCE was a mandatory session during the obstetrics and gynecology clerkship. Students received immediate feedback and optional individual reviews with clerkship directors. Students completed a nonmandatory survey at the end to describe their experience. SPIKES protocol student responses (i.e., proportion of correct responses) from in-person and remote SP versions were compared. **Results:** Between July 2019 and March 2020, 79 students completed the in-person OSCE. Between July 2020 and June 2021, 149 students completed the remote SP encounter OSCE. Students who participated in the remote versus the in-person OSCE were more likely to admit their lack of knowledge when not equipped ($p = .02$), be seated during the encounter ($p = .03$), show listening body language ($p = .13$), assess the SP's perception ($p = .19$) and understanding ($p = .20$), and correct the SP's misunderstandings ($p = .14$). Of 84 students from eight rotations, including both in-person and remote formats, 99% believed learning objectives were clear, 91% felt preparation material was adequate, 95% thought the instructor summarized important points, 97% learned something in caring for gynecological patients, and 96% perceived the OSCE to be a worthwhile educational experience. **Discussion:** The remote OSCE was well received by students. Breaking bad news virtually met assessment goals. Telemedicine training should be incorporated into medical school curricula.

Keywords:
Telemedicine, Breaking Bad News, SPIKES Protocol, Miscarriage, Case-Based Learning, Clinical Skills Assessment/OSCEs, OB/GYN, Standardized Patient, Telehealth

Educational Objectives

By the end of this session, students will be able to:

1. Obtain a focused history from a standardized patient experiencing a miscarriage.
2. Clinically assess a standardized patient having a miscarriage.
3. Engage a standardized patient in discussion of management options for a miscarriage.
4. Demonstrate professionalism in the domains of respect and compassion when breaking bad news.

Citation:
Prasad L, Hockstein S, Safdieh JE, Harvey K, Christos PJ, Kang Y. An objective structured clinical exam on breaking bad news for clerkship students: in-person versus remote standardized patient approach. *MedEdPORTAL*. 2023;19:11323.
https://doi.org/10.15766/mep_2374-8265.11323

Introduction

Early pregnancy loss, also called miscarriage or spontaneous abortion, occurs in 10% of clinically recognized pregnancies. Approximately 80% of cases occur within the first trimester.[1] This is a diagnosis seen in both fields of obstetrics and gynecology. Understandably, miscarriage can be distressing for the patient and requires more than a test results discussion. The consultation calls for communication that incorporates patience and empathy. In addition, providing counseling on management options including observation, medicine, or surgery is warranted. The Association of Professors of Gynecology and Obstetrics includes spontaneous abortion as an educational objective of the clerkship curriculum.[2]

SPIKES (setting up interview, assessing patient perception, invitation, giving knowledge and information to patient, assessing patient emotions with empathic responses, and summary and strategy), a mnemonic that organizes the steps in delivering bad news, is commonly used in medical education.[3] SPIKES

Copyright © 2023 Prasad et al. This is an open-access publication distributed under the terms of the Creative Commons Attribution license.

provides a six-step approach useful for a learner interviewing a patient, delivering results, and providing emotional support. During clerkship, a medical student may observe a health care provider breaking bad news in a clinical setting, but it is unlikely that a student would lead this discussion.

Standardized patient (SP) sessions have enhanced learner interaction and procedural skills in clinical fields, including obstetrics and gynecology.[4-6] Examples of breaking bad news communication curricula that utilize role-play and SPs are readily available.[7,8]

The COVID-19 pandemic required health care systems to adapt patient care delivery models. Consequently, virtual care has become progressively widespread and valuable.[9] For example, telemedicine allows for an initial assessment of miscarriage through history taking alone to guide management decisions preceding the physical exam. The American Medical Association has recommended telemedicine training be incorporated into medical education since most students will experience telemedicine at some point in their career.[10]

We developed a formative exercise as part of the obstetrics and gynecology clerkship curriculum in response to the COVID-19 pandemic. Given constraints on in-person activities, we converted our miscarriage objective structured clinical exam (OSCE) to a remote SP encounter. Participation in simulation through role-play with SPs allowed students to practice delivering news of a miscarriage case and to discuss findings and management plans in a remote encounter. Aligned with domains III and IV of the AAMC telehealth competencies,[11] the exercise included an analysis of communication and history-taking skills. The SPIKES protocol was used to assess students' rapport with patients through eye contact and body language, as well as their approach in evaluating and managing a miscarriage case.[11] We compared results between the previous in-person and remote SP encounter groups to evaluate the impact on student performance of breaking bad news in a virtual setting.

Methods

Target Audience

Medical students at Weill Cornell Medicine participate in a 6-week obstetrics and gynecology clerkship rotation. In July 2020, this was shortened to 4 weeks due to the COVID-19 pandemic. As part of the modified curriculum, the miscarriage OSCE was converted into a remote SP format. Students received an OSCE overview during orientation and were emailed instructions 3 days prior to the exercise. This information was available on their online learning website from day one of the rotation (Appendix A). For OSCE preparation, students were provided with three articles on early pregnancy loss[1,12,13] and the SPIKES protocol,[3] accessible online throughout the rotation. Students could view the patient note (Appendix B) and post-follow-up exercise (Appendices C and D) online ahead of the session. During their preclinical training, students had participated in small-group sessions and informal role-play focusing on breaking bad news. Students received limited formal training on the approach to a telemedicine visit in the curriculum.

SP Preparation

SPs were referred by other SPs who had the required acting and teaching experience. The clerkship director and SP educator collaborated to create the OSCE script for SPs. Interested SPs received case details so they could make an informed decision. Thirteen SPs accepted the role. They spent 3 hours training with the SP educator during which they reviewed standardization and strategies to manage unanticipated learner questions and behavior. They practiced providing feedback and learned how to complete assessments. SPs were prepared through repeated practice of the case.[14] They received the SP training guide (Appendix E), which included case details, instruction, expected learner responses, and the patient note answer key. On the OSCE day, participating SPs met with the SP educator 30 minutes before students arrived. They received updates, asked questions, and discussed OSCE-related issues. Following the session, a debrief with the SP educator was held to give SPs time to derole.

Before conversion to the remote OSCE, SPs received a refresher session about the case. Zoom etiquette regarding punctuality, internet connection, lighting, privacy, background, microphone muting, and eating or drinking during the encounter was reviewed. SPs received webcams and were taught on correct positioning for effective eye contact. The day before, SPs received the Zoom link and login email, and all used computers at home as they had internet access.

Patient Encounter

Introduction: Seven to 10 clerkship students were on rotation every 4 weeks. The OSCE was a mandatory assignment scheduled on a set day. Students joined remotely from a location with privacy and an internet connection. Two OSCE sessions were run one after the other, each lasting 45 minutes. Students were divided between these groups. For example, if there were seven students, four were in the first group and three in the following. One SP was assigned to two students, one from each group, for the day. One SP role-played for

Copyright © 2023 Prasad et al. This is an open-access publication distributed under the terms of the Creative Commons Attribution license.

both 45-minute sessions back to back, with a 5-minute break in between.

Students convened on Zoom in the main room. Here, the SP educator described OSCE steps. The scenario (Appendix A) portrayed a patient in the emergency room. After delayed menses, the patient had a positive home pregnancy test. Soon after, she experienced heavy bleeding and pelvic pain and was instructed by her gynecologist's office to go to the emergency room, where the SP interacted remotely with the learner, who was at a separate location.

The OSCE evaluator then met with the students. They reiterated OSCE steps and discussed the value of this opportunity to practice breaking bad news as well as the importance of developing this skill. The evaluator reviewed history-taking points, including use of open-ended questions, avoiding jargon, and how to be efficient. Evaluation included observation of the student-SP interaction, history-taking assessment, clinical decision-making, diagnosis, and management plan delivery. The evaluator had control on Zoom to move between breakout rooms during the session in order to observe all student-SP interactions simultaneously. A technician moved students between the main and breakout rooms. Students received an on-screen warning message when 2 minutes were left for each step.

Steps:

- The SP educator shared the first computer-screen door note with students in the main room (Appendix F).
- Initial encounter: Each student was moved to their individual breakout room to meet with their designated SP. They obtained a history and communicated relevant system exams and pelvic exam steps that would be performed in person. (15 minutes)
- Students were moved back to the main room where they received the second computer-screen door note indicating pelvic exam findings, transvaginal ultrasound, and blood test results (Appendix G). Here, they completed the computer patient note (Appendix B). (10 minutes)
- Follow-up: Students gathered their thoughts, returned to the breakout room with their SPs, delivered the diagnosis, and provided counseling and a management plan. (10 minutes)
- Students returned to the main room to complete the post-follow-up exercise (Appendices C and D). (5 minutes)
- Feedback: Students were moved back to their breakout rooms with their SPs for performance feedback. SPs used answers from the SPIKES protocol (Appendix H), history checklists (Appendix I), and the SP training guide (Appendix E) to provide students with an individualized and detailed assessment of their performance.
- The OSCE evaluator reconvened with students on Zoom to review answers for the patient note (Appendix E) and post-follow-up exercise (Appendices C and D), engage in discussion, and provide feedback on performance and comfort level during the encounter.

Learner Assessment

Students received verbal feedback from SPs immediately after the session. An assessment of their history-taking skills and aptitude to formulate an accurate diagnosis and management plan was accomplished through observation by the OSCE evaluator, and feedback was provided at the debrief. Also, history-taking and communication skills were evaluated by SPs, who completed the history checklist (Appendix I) and SPIKES protocol questionnaire (Appendix H), a psychometrically valid survey. Each SPIKES question allowed for a yes or no response, and yes was the correct response for each question. The proportion of all students in a given rotation who answered each SPIKES question correctly was recorded for each question.

Additionally, students' answers on the post-follow-up exercise (Appendices C and D) and performance evaluation by SPs through the history checklist (Appendix I) were tabulated into numeric assessments and were available for optional individual review with clerkship directors. Patient note answers (Appendix E) were also available for discussion; however, they were not scored numerically. These scores and answers were not included in the data analysis.

OSCE Evaluation

Students provided informal feedback to the OSCE evaluator at the session debrief. They could opt to complete and submit a five-question survey rating the clarity of the learning objectives, preparatory material quantity, instructor's important points summary, exercise content utility and future patient care relevance, and educational experience (Appendix J). Students could also add free comments. The college formal clerkship evaluation was another platform where students provided feedback.

Data Analysis

The proportion of correct responses for each rotation, as defined above, was averaged across six rotations in the pre-COVID period (nine to 17 students per rotation) and 12 rotations in the post-COVID period (eight to 17 students per rotation). The

Copyright © 2023 Prasad et al. This is an open-access publication distributed under the terms of the Creative Commons Attribution license.

average was weighted by rotation size and calculated for each SPIKES question separately. An exploratory statistical analysis was carried out for hypothesis-generating purposes. A two-sample *t* test was used to compare the mean proportion correct for each SPIKES question (i.e., sat down, showed listening body language, etc.) between the pre-COVID time period (six weighted mean proportions) and post-COVID time period (12 weighted mean proportions). We considered *p* values less than .05 statistically significant. Any *p* value between .06 and <.20 was considered a nonsignificant trend since the sample size of the mean proportions correct was very small in the two time-period cohorts ($N = 6$ and $N = 12$) and, thus, analyses were considered likely to be underpowered. All analyses were performed in IBM SPSS Statistics version 27.

Results

Seventy-nine students completed the pre-COVID (in-person) OSCE during academic year 2019-2020 between July 2019 and March 2020. One hundred forty-nine students completed the post-COVID (remote) OSCE during academic year 2020-2021 between July 2020 and June 2021. There were fewer pre-COVID (in-person) groups ($N = 6$) because there were no in-person clerkship activities from April 2020 until July 2020 due to COVID. Also, the clerkship length was shortened from 6 to 4 weeks in the post-COVID (remote) groups, which increased rotation numbers to 12 in academic year 2020-2021.

The Table reflects mean proportion correct (weighted across six rotations pre-COVID and 12 rotations post-COVID) for each question on the SPIKES protocol assessment. There was no significant difference between the in-person and remote groups' performance while communicating with SPs about addressing their readiness to discuss test results, using understandable language during diagnosis discussion, allowing SPs to express emotions and responding with empathy, and discussing a management plan and asking SPs for questions. Students were more likely to admit a lack of knowledge when not equipped during the remote encounter compared to the in-person group ($p = .02$). A nonsignificant trend indicated students were more likely to assess SPs' understanding ($p = .20$) and perception ($p = .19$), show listening body language ($p = .13$), and correct SPs' misunderstandings ($p = .14$) also in the remote setting (Table).

Of eight rotations, there were 84 students who chose to complete the survey, including four pre-COVID ($N = 52$ students) and four post-COVID rotations ($N = 32$ students). Respectively, 98% and 100% agreed on clear learning objectives, 88% and 93% agreed on adequate preparation materials, 90% and 100% thought important points were summarized, 100% and 93% learned something useful in gynecological patient care, and 98% and 93% perceived the OSCE as a worthwhile educational experience.

Student survey free-response comments for the remote encounter included the following:

- "This was awesome! Thank you."
- "This was helpful and important to learn in an SP clinical setting because even in my short time on the rotation I saw a similar case with a resident."
- "So happy to have the opportunity to become involved in a remote OSCE!"

At the debrief with the OSCE evaluator, students described the remote encounter as emotional and felt uncomfortable breaking bad news because they had never done it before. The evaluator stated that the students' encounter was similar to their real telemedicine visits in that they had to learn how to connect, speak softly and empathetically, and allow for silence during conversation. The evaluator thought students overall displayed good body language and that, similar to doctors, some were more empathetic than others.

SPs stated that numerous students appeared uncomfortable while explaining the diagnosis during the in-person and remote OSCEs. Students who struggled frequently used noncommittal jargon in an attempt to minimize the intensity of the news. SPs collectively observed that while delivering news in either format, a majority of students adhered to the SPIKES steps. However, SPs noted that silence was easier on Zoom and that some students expressed empathy better than others. A few started crying during the remote encounter.

Discussion

The remote SP encounter OSCE accomplished curriculum goals. Students had a virtual simulated clinical experience and were evaluated on their knowledge, communication skills, and ability to exhibit empathy and compassion while delivering bad news. This platform provided data to compare with students' previous in-person OSCE performance.

Telemedicine is useful for the delivery of a sensitive diagnosis such as miscarriage and can prevent delay in management. Our activity gave students an opportunity to practice an OSCE case in a realistic learning environment, and overall, students provided positive feedback. Remote SP encounter survey results showed that students believed the learning objectives were clear (100%),

Copyright © 2023 Prasad et al. This is an open-access publication distributed under the terms of the Creative Commons Attribution license.

Table. Mean Difference in SPIKES Protocol Proportion Correct Between Pre- and Post-COVID Cohorts

	Proportion Correct *M* (*SD*)			
**OSCE SPIKES Protocol Elements**	**Pre-COVID**[a]	**Post-COVID**[b]	***M* Difference (95% CI)**	***p***
Setting up interview				
Sat down	91.8 (6.9)	100.0 (0.0)	8.2 (1.0 to 15.4)	.03
Showed listening body language	95.4 (5.8)	98.7 (3.1)	3.3 (−1.1 to 7.7)	.13
Assessing patient perception				
Asked me what I knew or had been told about results	52.0 (11.1)	60.5 (13.0)	8.6 (−4.6 to 21.7)	.19
Invitation				
Asked me if I was ready to talk about the exam results	74.9 (12.3)	75.0 (10.7)	0.0 (−11.9 to 11.9)	.99
Giving knowledge and info to the patient				
Used language that I could understand	81.7 (8.5)	77.3 (12.0)	−4.4 (−16.1 to 7.2)	.43
Periodically assessed my understanding	72.3 (16.0)	81.6 (12.9)	9.3 (−5.4 to 24.1)	.20
Corrected misunderstandings	83.3 (10.8)	90.6 (8.9)	7.3 (−2.8 to 17.5)	.14
Admitted to lack of knowledge when not equipped	68.1 (10.9)	83.6 (11.6)	15.5 (3.5 to 27.6)	.02
Assessing patient's emotions with empathic responses				
Gave me the opportunity to express my emotions	88.0 (6.1)	84.8 (10.8)	−3.1 (−13.3 to 7.0)	.52
Responded to me with empathy	92.9 (9.2)	89.7 (10.3)	−3.2 (−13.8 to 7.4)	.53
Summary and strategy				
Discussed a management plan	90.9 (5.8)	91.7 (7.7)	0.8 (−6.8 to 8.4)	.83
Asked me if I had any questions	94.1 (5.4)	96.5 (5.8)	2.5 (−3.6 to 8.5)	.40

[a]$N = 6$.
[b]$N = 12$.

the preparation material was adequate (93%), they learned something useful (93%), the experience was worthwhile (93%), and important points were well summarized by the evaluator (100%).

Using a remote format allowed students from multiple clinical sites to participate without the need to physically convene at one campus. In comparison with the in-person OSCE, the remote encounter group had similar scores for 50% of the domains generated through the SPIKES protocol checklist completed by the SPs. Students who participated in the remote encounter were more likely than those in the in-person group to admit their lack of knowledge when not equipped. Other checklist questions showed a nonsignificant trend. Students were more likely to show listening body language, correct the SPs' misunderstandings, and assess the SPs' perception and understanding during the remote encounter. The results for the item about being seated during the encounter were likely a function of the remote encounter format. The same SPs participated in the in-person and remote encounter OSCEs.

Results indicating that the remote encounter might be the more effective format for certain areas may merit additional investigation. It could be that for early trainees, a technology platform creates a safe distance, making it easier for a student to admit lack of knowledge and to correct misunderstandings. Also fascinating is that the SPs' perception of students showing better listening body language may be in part because Zoom forces visual focus on the person talking. An earlier study showed that patients expecting bad news wanted to learn results as quickly as possible and were more focused on message content than supportive aspects of communication, such as a hand on the shoulder.[15] Virtual visits reassure patients that providers are involved in their care, improving a patient's (or SP's) assessment of their provider.[16] Even in the business industry and remote working, visual is more effective compared to audio. Visual communication through videoconferencing builds camaraderie, productivity, and overall collaboration.[17] Telemedicine may lend itself to facilitating more advanced and uncomfortable conversations.

Students reported they felt uncomfortable breaking bad news. Data show that even during residency, a majority feel insufficient in conveying bad news to patients.[18] Good communication skills are important for an effective doctor-patient relationship and contribute to improved patient satisfaction and fewer lawsuits.[19] However, significant gaps in how physicians deliver bad news exist, and doctors acknowledge their lack of training.[18,20] In one study, only 47% of patients were pleased with how bad news was delivered to them, and this influenced their decisions regarding medical treatment and changing physicians in charge.[21] The need to continue teaching this skill at different medical training levels is recognized.

Limitations included our having a small sample size. At best, our data suggest the remote encounter is noninferior to the in-person OSCE as an effective platform for breaking bad news. However, not all AAMC telehealth competencies have been

Copyright © 2023 Prasad et al. This is an open-access publication distributed under the terms of the Creative Commons Attribution license.

incorporated in this activity, so it is not an actual telemedicine simulation.

Other limitations included the following: There was back and forth between virtual rooms. Also, the OSCE evaluator moved between breakout rooms and occasionally would miss key portions of students' performance. While Zoom allows for recordings that can be viewed afterward, we suggest that sites replicating this project utilize three evaluators to eliminate concerns for incomplete assessments. We have modified our remote encounter so that students move fewer times between rooms (Appendix K).

We did not include survey questions specifically about the remote experience, which we intend to modify. Although SPs' feedback is reflected on the SPIKES checklist, in the future we can also document their verbal feedback to share with students and enhance their learning experience. Immediate post-OSCE feedback from SPs about students' empathy and rapport building has been well received by students in the literature.[22]

This has been a useful exercise for students to practice delivering bad news in a virtual format. Our primary focus was to evaluate students' communication skills and expression of empathy and compassion. As virtual visits continue to become well accepted in medicine, addressing sensitive topics via telemedicine should be incorporated into the medical education curriculum at our institution. We will continue the OSCE as a remote encounter in the obstetrics and gynecology clerkship and incorporate AAMC telehealth competency domains into the structure to create a telemedicine simulation. Adaptations to the traditional SPIKES model, such as using more exaggerated body language and verbally confirming an alternative way to contact the SP in event of technologic failure, will also be included in our virtual training.[23]

Appendices

A. SP Case.docx
B. Patient Note.pdf
C. Post-Follow-up Exercise.pdf
D. Post-Follow-up Exercise Answer Key.docx
E. SP Training Guide.pdf
F. Door Note (First Encounter).pdf
G. Door Note (Second Encounter).pdf
H. SPIKES Protocol Checklist.pdf
I. History Checklist.pdf
J. Five-Question Survey.pdf
K. OSCE Instructions.pdf

All appendices are peer reviewed as integral parts of the Original Publication.

Lona Prasad, MD: Assistant Professor and Director, Clerkship for Undergraduate Medical Education, Department of Obstetrics and Gynecology, New York-Presbyterian/Weill Cornell Medicine; ORCID: https://orcid.org/0000-0002-2549-4073

Steven Hockstein, MD: Associate Professor and Associate Director, Clerkship for Undergraduate Medical Education, Department of Obstetrics and Gynecology, New York-Presbyterian/Weill Cornell Medicine

Joseph E. Safdieh, MD: Professor and Vice Chair of Education, Department of Neurology, New York-Presbyterian/Weill Cornell Medicine

Kevaughn Harvey, MFA: Clinical Skills Coordinator, New York-Presbyterian/Weill Cornell Medicine

Paul J. Christos, DrPH: Associate Professor of Research in Population Health Sciences, Division of Biostatics, New York-Presbyterian/Weill Cornell Medicine

Yoon Kang, MD: Associate Professor, Medical Education, Office of Medical Education, Weill Cornell Medicine

Disclosures
None to report.

Funding/Support
Dr. Paul J. Christos was partially supported by a grant from the Clinical and Translational Science Center at Weill Cornell Medicine Medical College (1-UL1-TR002384-01).

Ethical Approval
The Weill Cornell Medicine Medical College Institutional Review Board approved this project.

References

1. ACOG Practice Bulletin no. 200: early pregnancy loss. *Obstet Gynecol*. 2018;132(5):e197-e207. https://doi.org/10.1097/AOG.0000000000002899
2. *APGO Medical Student Educational Objectives*. 11th ed. Association of Professors of Gynecology and Obstetrics; 2019. Accessed May 1, 2023. https://cdn.ymaws.com/apgo.site-ym.com/resource/resmgr/apgo-11th-ed-mso-book.pdf
3. Baile WF, Buckman R, Lenzi R, Glober G, Beale EA, Kudelka AP. SPIKES—a six-step protocol for delivering bad news: application to the patient with cancer. *Oncologist*. 2000;5(4):302-311. https://doi.org/10.1634/theoncologist.5-4-302

Copyright © 2023 Prasad et al. This is an open-access publication distributed under the terms of the Creative Commons Attribution license.

4. May W, Park JH, Lee JP. A ten-year review of the literature on the use of standardized patients in teaching and learning: 1996–2005. *Med Teach*. 2009;31(6):487-492. https://doi.org/10.1080/01421590802530898
5. Lane C, Rollnick S. The use of simulated patients and role-play in communication skills training: a review of the literature to August 2005. *Patient Educ Couns*. 2007;67(1-2):13-20. https://doi.org/10.1016/j.pec.2007.02.011
6. Rodriguez AN, Fisher J, Broadfoot K, Hurt KJ. A challenging obstetric communication experience for undergraduate medical education using standardized patients and student self-reflection. *MedEdPORTAL*. 2015;11:10121. https://doi.org/10.15766/mep_2374-8265.10121
7. van Zuilen M, Caralis P, Granville L. Breaking bad news: a small group session teaching communication skills for delivering bad news. *MedEdPORTAL*. 2013;9:9604. https://doi.org/10.15766/mep_2374-8265.9604
8. Cvengros JA, Behel JM, Finley E, Kravitz R, Grichanik M, Dedhia R. Breaking bad news: a small-group learning module and simulated patient case for preclerkship students. *MedEdPORTAL*. 2016;12:10505. https://doi.org/10.15766/mep_2374-8265.10505
9. Fact sheet: the future of telehealth—protecting patient access to care. American Hospital Association. 2020. Accessed May 1, 2023. https://www.aha.org/fact-sheets/2020-07-30-fact-sheet-future-telehealth-protecting-patient-access-care
10. AMA encourages telemedicine training for medical students, residents. American Medical Association. June 15, 2016. Accessed May 1, 2023. https://www.ama-assn.org/ama-encourages-telemedicine-training-medical-students-residents
11. Association of American Medical Colleges. *Telehealth Competencies Across the Learning Continuum*. Association of American Medical Colleges; 2021. Accessed May 1, 2023. https://store.aamc.org/downloadable/download/sample/sample_id/412/
12. Doubilet PM, Benson CB, Bourne T, Blaivas M; Society of Radiologists in Ultrasound Multispecialty Panel on Early First Trimester Diagnosis of Miscarriage and Exclusion of a Viable Intrauterine Pregnancy. Diagnostic criteria for nonviable pregnancy early in the first trimester. *N Engl J Med*. 2013;369(15):1443-1451. https://doi.org/10.1056/NEJMra1302417
13. Griebel CP, Halvorsen J, Golemon TB, Day AA. Management of spontaneous abortion. *Am Fam Physician*. 2005;72(7):1243-1250.
14. Lewis KL, Bohnert CA, Gammon WL, et al. The Association of Standardized Patient Educators (ASPE) Standards of Best Practice (SOBP). *Adv Simul (Lond)*. 2017;2:10. https://doi.org/10.1186/s41077-017-0043-4
15. Krishnan N, Fagerlin A, Skolarus TA. Rethinking patient-physician communication of biopsy results—the waiting game. *JAMA Oncol*. 2015;1(8):1025-1026. https://doi.org/10.1001/jamaoncol.2015.2334
16. Powell RE, Henstenburg JM, Cooper G, Hollander JE, Rising KL. Patient perceptions of telehealth primary care video visits. *Ann Fam Med*. 2017;15(3):225-229. https://doi.org/10.1370/afm.2095
17. Roy BD. How to overcome the communication barriers for remote employees. *Vantage Circle* blog. April 19, 2020. Updated April 27, 2023. Accessed May 1, 2023. https://blog.vantagecircle.com/communication-blocks-for-remote-working-employees/
18. Orgel E, McCarter R, Jacobs S. A failing medical educational model: a self-assessment by physicians at all levels of training of ability and comfort to deliver bad news. *J Palliat Med*. 2010;13(6):677-683. https://doi.org/10.1089/jpm.2009.0338
19. Huntington B, Kuhn N. Communication gaffes: a root cause of malpractice claims. *Proc (Bayl Univ Med Cent)*. 2003;16(2):157-161. https://doi.org/10.1080/08998280.2003.11927898
20. Warrier V, Pradhan A. A narrative review of interventions to teach medical students how to break bad news. *Med Sci Educ*. 2020;30(3):1299-1312. https://doi.org/10.1007/s40670-020-01015-x
21. Sobczak K, Leoniuk K, Janaszczyk A. Delivering bad news: patient's perspective and opinion. *Patient Prefer Adherence*. 2018;12:2397-2404. https://doi.org/10.2147/PPA.S183106
22. Newcomb AB, Duval M, Bachman SL, Mohess D, Dort J, Kapadia MR. Building rapport and earning the surgical patient's trust in the era of social distancing: teaching patient-centered communication during video conference encounters to medical students. *J Surg Educ*. 2021;78(1):336-341. https://doi.org/10.1016/j.jsurg.2020.06.018
23. Vitto C, Del Buono B, Daniel L, Rivet E, Cholyway R, Santen SA. Teaching toolbox: breaking bad news with virtual technology in the time of COVID. *J Cancer Educ*. 2022;37(5):1429-1432. https://doi.org/10.1007/s13187-021-01975-7

Received: August 16, 2022
Accepted: April 6, 2023
Published: July 21, 2023

Copyright © 2023 Prasad et al. This is an open-access publication distributed under the terms of the Creative Commons Attribution license.
